# Analysis of the application effect of the Clark comfortable nursing approach in hemodialysis patients with end stage renal failure

**DOI:** 10.1080/0886022X.2024.2423011

**Published:** 2024-11-14

**Authors:** Jiankai Li, Yujie Lin, Linlin Wang, Qinglan Wang, Qing Wu

**Affiliations:** Department of Hemodialysis, Shanghai University of Traditional Chinese Medicine Affiliated Shuguang Hospital, Shanghai, China

**Keywords:** Clark comfortable nursing, Chronic kidney disease, hemodialysis, self-care ability, self-burden, treatment adherence

## Abstract

**Objective:**

This study observed the effects of the Clark comfortable nursing approach on self-care ability, self-burden, treatment adherence, quality of life, and complications in hemodialysis patients with end stage renal failure (ESRF).

**Methods:**

Eighty-two patients with ESRF receiving hemodialysis treatment were included and allocated into control and intervention groups. The control group received conventional nursing care, while the intervention group received the Clark comfortable nursing approach. The self-care ability, self-burden, treatment adherence, quality of life scores before and after the nursing intervention, and the occurrence of complications in both groups were compared.

**Results:**

After the intervention, the intervention group showed higher scores in each dimension and the total score of the Exercise of Self-Care Agency Scale compared to the control group. Both groups exhibited improvements in various scores and total scores; however, the intervention group had lower scores overall than the control group. Additionally, the intervention group had higher scores in diet, water intake, medication, and dialysis regimen. Additionally, both groups had significantly higher scores in all dimensions of the quality-of-life scale post-intervention, with the intervention group demonstrating markedly higher scores in all dimensions. The total incidence of complications in the intervention group was 9.76%, which was lower than the 29.27% observed in the control group.

**Conclusion:**

The Clark comfortable nursing approach applied to hemodialysis patients with ESRF can enhance self-care ability, improve quality of life, increase treatment adherence, and reduce the incidence of hemodialysis-related complications. This model is worthy of clinical promotion.

## Introduction

Chronic kidney disease (CKD) is a common and significant condition worldwide, associated with substantial mortality and morbidity [1]. CKD results from impaired kidney function, an organ crucial for the metabolism, filtration, and excretion of compounds [2]. It can accelerate cardiovascular disease, elevate the risk of infection, and contribute to anemia, bone disease, and other complications that jointly enhance the risk of premature death [3,4]. CKD also interferes with the physiological and biological mechanisms of the body, including processes associated with its liquid electrolyte and pH balance, toxin and waste excretion, blood pressure regulation, vitamin D metabolism, and hormone regulation [5]. Patients with this condition are asymptomatic most of the time, and the typical complications of renal insufficiency occur only in advanced stages. Treatment can be conservative (patients without indication for dialysis, usually those with a glomerular filtration rate of more than 15 mL/min) or alternative (hemodialysis, peritoneal dialysis, and kidney transplantation) [6].

Emotional distress often occurs during dialysis and is characterized by extreme apprehension, anxiety, despair, and/or depression due to the patient’s perceived inability to cope with the challenges and demands of dialysis life [7]. About 40% of hemodialysis patients suffer from ‘moderate’ to ‘extreme’ itchiness, which is associated with decreased quality of life, poor sleep, depression, and worsened clinical outcomes [8]. These problems seriously affect their quality of life and adherence to treatment. Given the direct influence of symptoms on patient health, the diagnosis and management of symptoms are essential focuses of patient-centered nursing [9]. Currently, although the conventional nursing model meets the physiological needs of patients to a certain extent, it still needs to be strengthened in terms of alleviating the emotional and symptomatic burdens. Therefore, it is particularly important to explore a more comprehensive and personalized care model. Clark, an American nursing expert who improves nursing procedures based on the theory of ‘comfortable nursing’, notes that his program can effectively help patients establish their awareness of self-management [10]. The Clark comfortable nursing approach involves assessing patients’ psychological status before dialysis, where nurses and patients work together to identify existing issues and develop solutions and measures to address them. The nursing team members devise appropriate intervention strategies for the patients, and after jointly analyzing the pros and cons, the patients themselves choose the interventions. Subsequently, with the patient as the center, the nursing staff develops a Clark comfortable nursing plan, using time as the abscissa and nursing content as the ordinate, to design a care pathway table for patients with end-stage renal disease, which includes measures for diet, medication, exercise, health education, and emotional management. Additionally, the nursing team members monitor the patients’ self-management daily and conduct weekly evaluations with the patients to analyze and rectify based on the results before moving to the next cycle [11]. Data indicate that applying the Clark comfortable nursing approach in long-term hemodialysis patients with uremia yields satisfactory results. It effectively improves psychological resilience, reduces perceived self-burden, and enhances self-management levels and quality of life [12]. For patients with ESRF, this model significantly boosts self-care abilities, enhances hope, and improves overall survival quality [13]. This indicates that the Clark comfortable nursing approach is a comprehensive and personalized approach that aims to achieve holistic physical, psychological, social, and spiritual comfort and rehabilitation by promoting self-management and active participation. This approach not only focuses on patients’ physical health but also emphasizes the harmony of their psychological and social relationships and spiritual fulfillment. Therefore, based on the aforementioned characteristics of the Clark comfortable nursing approach, we have reason to believe that its application in hemodialysis patients with chronic kidney disease may markedly improve patients’ self-care ability, psychological state, quality of life, and adherence to treatment. This study’s development and implementation of the Clark comfortable nursing approach were tailored to the patients’ actual situations and the standards of our hospital. To accurately assess the effectiveness of the Clark comfortable nursing approach in alleviating the symptomatic burden of hemodialysis patients, the following evaluation measures will be employed in this study: the Exercise of Self-Care Agency Scale (ESCA), the Self-Perceived Burden Scale (SPBS), treatment adherence assessment, and the 36-Item Short Form Health Survey (SF-36). The ESCA is used to assess patients’ self-care ability. Through this scale, one can understand patients’ self-management capabilities in daily life [14]. This is significant for evaluating the improvement in patients’ self-care ability through the Clark comfortable nursing approach. The SPBS measures patients’ perceived burden. With this scale, one can ascertain the degree to which patients feel burdened by their illness and treatment [15]. This is crucial for evaluating the Clark comfortable nursing approach’s effectiveness in alleviating patients’ psychological burden. Treatment adherence assessment is a critical indicator of whether patients follow medical advice for treatment. It can be assessed by recording patients’ dialysis protocols, medication adherence, dietary control, etc. Good treatment adherence is vital for improving dialysis treatment outcomes and patients’ quality of life. The 36-Item Short Form Health Survey scale is a widely adopted tool for assessing quality of life [16]. This scale covers various aspects such as physical functioning, social functioning, and mental health, providing a comprehensive assessment of patients’ quality of life. Through the 36-Item Short Form Health Survey scale, one can understand the improvement in patients’ quality of life through the Clark comfortable nursing approach. By combining multiple assessment measures, this study aimed to evaluate the effects of this approach on self-care ability, perceived self-burden, treatment adherence, quality of life, and the occurrence of complications in hemodialysis patients with ESRF.

## Materials and methods

### Ethical approval

This study was approved by the Ethics Committee of Shanghai University of Traditional Chinese Medicine Affiliated Shuguang Hospital (approval number: 20220306). Written informed consent was obtained from all the subjects.

### Participants

Patients with ESRF on hemodialysis who underwent hemodialysis treatment at Shanghai University of Traditional Chinese Medicine Affiliated Shuguang Hospital between June 2022 and July 2023 were selected. Inclusion criteria: those diagnosed with ESRF based on blood, urine, imaging, and kidney biopsy findings; those who met the indications for hemodialysis and had been on hemodialysis for ≥ 3 months; and those whose conditions were more stable. The exclusion criteria were as follows: serious dysfunction of the heart, liver, lungs, or other vital organs; severe renal failure or unstable condition; mental illness or cognitive disorders; serious infections or malignant tumors; and pregnancy and lactation.

Based on the inclusion and exclusion criteria, 82 patients were selected. In accordance with the random number table method, 82 patients were randomly allocated to the control and intervention groups (41 cases each). There was no significant difference in the general information of the patients in the two groups (*p* > 0.05), indicating that they were comparable (Table 1).

**Table 1.** Comparison Of general information between two groups of patients.

	Control group (*n* = 41)	Intervention group (*n* = 41)	χ2/t	*P*
Age (years)	55.29 ± 4.57	54.76 ± 4.82	0.517	0.606
Gender			0.198	0.656
Male	22 (53.66)	24 (58.54)		
Female	19 (46.34)	17 (41.46)		
BMI (kg/m2)	22.68 ± 2.14	22.57 ± 2.09	0.237	0.814
Average hemodialysis time (years)	2.46 ± 1.14	2.42 ± 1.07	0.16	0.873
Primary diseases			1.022	0.796
Diabetes nephropathy	12 (29.27)	10 (24.39)		
Chronic glomerulonephritis	16 (39.02)	18 (43.90)		
Hypertensive nephropathy	10 (24.39)	8 (19.51)		
Other	3 (7.32)	5 (12.20)		
Underlying diseases				
Hypertension	18(43.90)	15(36.59)	0.456	0.499
Diabetes mellitus	16(39.02)	12(29.27)	0.868	0.352
Hyperlipidemia	10(24.39)	8(19.51)	0.285	0.594
Dialysis modality			0.617	0.735
Arteriovenous endovascular fistula	27	25		
Artificial vessels	5	4		
Catheter with polyester sleeve with tunnel	9	12		

### Methods

All patients received routine hemodialysis treatment according to the nephrologists’ prescription, with sessions lasting 4 h, three times per week. During the treatment period, the patients’ blood glucose and blood pressure levels were monitored and managed, and they were administered folic acid, calcium carbonate, iron, erythropoietin, and other symptomatic treatments. To avoid contamination between groups, patients in the control group and those in the intervention group were assigned to different dialysis rooms for their treatments.

Control group: patients in this group received routine nursing care. (1) The responsible nurse provided basic health education, covering topics such as the disease, the principles of dialysis treatment, the duration and frequency of dialysis, and related adverse reactions. Nursing staff were encouraged to communicate more with patients, demonstrating empathy and patience while also understanding changes in the patient’s emotional state. Additionally, they were to encourage patients to cooperate with their treatment and maintain communication with the patient’s family, advising them to offer care and psychological support. Patients were instructed on daily self-management, which included a reasonable diet (emphasizing adequate calorie intake while adhering to a low-sodium, low-phosphorus, low-potassium, high-quality protein diet, and ensuring proper hydration to maintain a balanced nutritional profile and enhance immunity), condition monitoring (daily checks of vital signs such as temperature, pulse, and respiration), and lifestyle modifications (ensuring sufficient sleep and rest, quitting smoking and drinking, exercising appropriately according to individual tolerance, and engaging in activities or work within their capabilities). (2) A conducive dialysis environment significantly impacts the patient’s condition and prognosis; therefore, nursing care included providing a quiet, comfortable, and clean dialysis environment while controlling temperature and humidity in the dialysis room. (3) The patient’s vital signs during dialysis were closely monitored, along with observations of the patient’s facial expressions and mental state. Complications such as hypotension, imbalance syndrome, hemorrhage, and pyrogenic reactions were promptly addressed as soon as they occurred. (4) Vascular access was regularly checked to prevent and address dislocations, blockages, and other abnormalities as soon as they were detected. (5) After treatment, patients were instructed to apply sufficient compression at the puncture site and to avoid injections, punctures, or invasive examinations for 2–4 h. Regular telephone follow-ups were conducted to assess the patient’s condition and provide guidance, and patients were advised to visit the hospital regularly for treatment or checkups

Intervention group: Patients in this group received the Clark comfortable nursing approach in addition to the standard care provided to the control group. (1) Formation of the Clark comfortable nursing team: The team leader was the head nurse, and the team members were nursing staff with more than three years of experience in the department. Regular lectures on knowledge related to the Clark comfortable nursing approach were held to provide relevant training to all team members. An assessment was arranged at the end of the training to ensure all team members could formally carry out nursing care after passing the assessment. (2) Formulation of the Clark comfortable nursing procedure: Solutions were developed by combining the challenges faced by patients with this disease during dialysis with the principles of the Clark comfortable nursing approach. This comprehensive approach resulted in a targeted nursing intervention program. (3) Implementation of the Clark comfortable nursing approach: ① Before the start of dialysis, nursing staff first assessed the patient’s psychological state using the Connor–Davidson Resilience Scale. Targeted psychological interventions were then carried out based on the assessment results. Staff explained the impact of negative emotions on the disease and overall quality of life. For patients experiencing severe negative emotions, one-on-one interviews were conducted to help regulate their feelings. Communication with patients involved understanding their daily exercise, diet, and medication, which were documented accordingly. Concerns were raised based on the patient’s condition, and discussions were held to develop improvement strategies. ② During dialysis, once a week, nursing staff also provided personalized health education using a combination of PowerPoint presentations, videos, and pictures to systematically convey essential information on chronic renal failure (etiology, risk factors, treatment options, and disease management), hemodialysis (principles, significance, common complications, and prevention methods), and the Clark comfortable nursing approach (definition, significance, and implementation methods). Patients were randomly asked about their understanding of the disease and hemodialysis; the results guided targeted corrections to enhance their knowledge. ③ The Clark comfortable nursing approach was tailored to each patient, incorporating a nursing pathway table based on the intervention program. A checklist was created so patients could mark tasks as completed, and a handout detailing care instructions was distributed. The table encompassed dietary content, exercise, arteriovenous fistula self-care, medication adherence, emotional counseling, health education, and more. For dietary self-care, nursing staff arranged meal plans to ensure adequate daily nutrient and calorie intake while reducing the risk of malnutrition. Patients were instructed to adhere strictly to medication prescriptions, and nursing staff communicated any unauthorized changes in medication or dosages to improve patient understanding of proper drug use.

For lifestyle self-care, after dialysis treatment, patients were encouraged to engage in walking, jogging, or other exercises based on their tolerance levels, with heart rate and blood pressure monitored before and after exercise. Patients were advised to ensure sufficient sleep, avoid staying up late, and quit smoking and drinking. Regarding psychological self-care, patients were encouraged to maintain a relaxed and happy mood by diverting their attention with favorite films, videos, or music and managing their emotions through positive thinking meditation or deep breathing exercises.

For arteriovenous fistula self-care, patients were instructed to keep the arm side of the fistula dry and clean. They also performed exercises three times a day using a rubberized grip ring and were instructed on how to self-examine the fistula for patency. Patients were also responsible for monitoring their own complications by observing vital signs such as pulse, blood pressure, and respiratory rate. They were instructed to report any symptoms such as weakness, fatigue, or rapid heartbeat to a nurse or doctor in a timely manner. Nursing staff were responsible for supervising patients in following the self-care plan, monitoring their adherence, and discussing implementation progress weekly to identify and address any deficiencies. Both groups received these interventions for three months.

### Observation indicators

Self-care ability: the ESCA was used to assess patients’ self-care ability before and after the intervention. The scale included four dimensions: self-concept, health cognitive level, self-care responsibility, and self-care skills, with a total of 43 items. The number of items in each dimension was 8, 17, 6, and 12, respectively, with a score of 0–4 for each item and a total score of 172. Higher scores indicated better self-care skills [14].

Self-burden: The SPBS was adopted to evaluate the self-perceived burden of both patient groups before and after the intervention. The scale included three dimensions: physical, emotional, and economic, with a total of 10 items. The 8th item was reverse scored, using a 1–5 point scale, resulting in a total score range of 10–50. Scores were positively correlated with self-burden [17].

Treatment adherence: The Hemodialysis Treatment Adherence Scale was employed to assess patients’ treatment adherence before and after the intervention. This scale included four dimensions: diet, fluid intake, medication, and dialysis regimen, with a total of 23 items. Each item was scored from 1 to 5, resulting in a total score range of 23–115 points, with higher scores indicating better patient adherence.

Quality of life: The quality of life of patients in both groups was evaluated before and after the nursing intervention using the 36-Item Short Form Health Survey scale. This scale included four dimensions: physical function, psychological function, social function, and material life. All scores were calculated using the percentage scoring method; higher scores indicated a better quality of life [18].

Complications: The occurrence of complications during the nursing intervention period was statistically analyzed and compared between the two patient groups, including bleeding, infection, catheter occlusion, hemodialysis-induced hypotension, and malnutrition. The nursing staff observed or documented the occurrence of complications through follow-up during the intervention period. Bleeding was defined as oozing at the puncture site or gastrointestinal bleeding. Infection primarily referred to vascular access and internal fistula infections, as well as catheter-related infections (such as those involving artificial blood vessels and deep venous catheterization) that occurred during hemodialysis. Catheter occlusion was indicated by the blockage of artificial blood vessels or deep venous catheters. Hemodialysis-induced hypotension mainly occurred during hemodialysis, characterized by a decrease in systolic blood pressure by ≥2 0 mmHg or a decrease in mean arterial pressure by ≥ 10 mmHg, accompanied by symptoms such as dizziness, pallor, nausea, and vomiting in patients.

### Statistical analysis

SPSS software (version 25.0; IBM SPSS Statistics, Chicago, IL, USA) was used to process the data, and the normality of the measured data was analyzed using the Kolmogorov–Smirnov test. Measurement data were expressed as mean ± standard deviation and analyzed using *t*-tests. Categorical data were expressed as n (%) and were analyzed using the Chi-square test. Differences were considered statistically significant at *p* < 0.05.

## Results

### Self-care abilities

Before the intervention, there were no significant differences in the ESCA scores for any dimension or the total score between the control and intervention groups (*p* > 0.05). After the intervention, the intervention group showed higher ESCA scores for each dimension as well as a higher total score compared to the control group (*p* < 0.05; Table 2).

**Table 2.** Comparison Of self-care abilities between two groups of patients.

	Control group (*n* = 41)	Intervention group (*n* = 41)	t	*P*
Before intervention				
Self-concept	17.68 ± 1.62	17.61 ± 1.70	0.200	0.842
Health cognitive level	30.71 ± 1.86	31.20 ± 1.62	1.267	0.209
Self-care responsibility	12.27 ± 1.12	12.56 ± 1.29	1.100	0.275
Self-care skills	28.59 ± 1.36	29.05 ± 1.48	1.475	0.144
Total score	89.24 ± 2.99	90.41 ± 2.81	1.827	0.071
After intervention				
Self-concept	26.54 ± 1.36*	28.88 ± 1.68*	6.942	< 0.001
Health cognitive level	45.10 ± 2.28*	48.66 ± 2.34*	6.98	< 0.001
Self-care responsibility	19.34 ± 1.32*	22.27 ± 1.45*	9.574	< 0.001
Self-care skills	39.39 ± 1.46*	42.68 ± 2.20*	7.988	< 0.001
Total score	130.37 ± 3.42*	142.49 ± 3.56*	15.730	< 0.001

Note: *p < 0.05 vs the same group before intervention.

### Self-burden

Before the intervention, no significant differences were observed in the physical, emotional, economic, or total SPBS scores between the control and intervention groups (*p* > 0.05). After the intervention, both groups showed improvements in various scores and total scores, and the intervention group had lower scores and total scores than the control group (*p* < 0.05; Table 3).

**Table 3.** Comparison Of self-burden between two groups of patients.

	Control group (*n* = 41)	Intervention group (*n* = 41)	t	*P*
Before intervention				
Physical	14.98 ± 2.17	15.00 ± 2.17	0.051	0.960
Emotional	14.51 ± 2.54	14.20 ± 2.38	0.583	0.561
Economic	4.27 ± 1.34	4.32 ± 1.47	0.157	0.876
Total score	33.76 ± 3.40	33.51 ± 3.23	0.333	0.740
After intervention				
Physical	7.44 ± 2.01*	5.88 ± 1.78*	3.722	< 0.001
Emotional	8.98 ± 1.99*	6.54 ± 1.63*	6.066	< 0.001
Economic	3.46 ± 0.95*	3.02 ± 0.82*	2.237	0.028
Total score	19.88 ± 3.33*	15.44 ± 2.86*	6.476	< 0.001

Note: *p < 0.05 vs the same group before intervention.

### Treatment compliance

Before the intervention, there was no significant difference in the scores for diet, water intake, medication, or dialysis regimen between the control and intervention groups (*p* > 0.05). After the intervention, the intervention group had higher scores than the control group in all aspects (*p* < 0.05; Table 4).

**Table 4.** Comparison Of treatment compliance between two groups of patients.

	Control group (*n* = 41)	Intervention group (*n* = 41)	t	*P*
Before intervention				
Diet	20.41 ± 2.51	20.17 ± 2.43	0.447	0.656
Drinking water	15.54 ± 2.17	15.49 ± 2.31	0.098	0.922
Medication	13.59 ± 2.09	13.20 ± 2.00	0.864	0.390
Dialysis regimen	12.05 ± 1.36	11.95 ± 1.45	0.315	0.754
After intervention				
Diet	24.59 ± 2.93*	28.56 ± 2.70*	6.384	< 0.001
Drinking water	18.37 ± 2.79*	22.12 ± 2.64*	6.263	< 0.001
Medication	17.59 ± 2.29*	21.78 ± 2.62*	7.714	< 0.001
Dialysis regimen	15.66 ± 1.62*	18.39 ± 1.48*	7.964	< 0.001

Note: *p < 0.05 vs the same group before intervention.

### Quality of life

Both groups had significantly higher scores in all dimensions of the quality-of-life scale after intervention compared to before intervention (*p* < 0.05), and the intervention group had markedly higher scores in all dimensions after intervention compared to the control group (*p* < 0.05; Table 5).

**Table 5.** Comparison Of quality of life between two groups of patients.

	Control group (*n* = 41)	Intervention group (*n* = 41)	t	*P*
Before intervention				
Physical function	55.63 ± 5.30	56.78 ± 5.47	0.963	0.338
Psychological function	55.37 ± 6.00	55.00 ± 5.87	0.279	0.781
Social function	56.51 ± 5.85	56.73 ± 5.53	0.175	0.862
Material life	54.27 ± 5.23	53.93 ± 5.18	0.297	0.767
After intervention				
Physical function	62.61 ± 6.18*	72.90 ± 7.61*	6.72	< 0.001
Psychological function	61.39 ± 6.85*	74.34 ± 8.20*	7.761	< 0.001
Social function	64.41 ± 6.50*	75.29 ± 7.52*	7.005	< 0.001
Material life	64.27 ± 6.78*	72.29 ± 7.17*	5.209	< 0.001

Note: *p < 0.05 vs the same group before intervention.

## Incidence of complications

The total incidence of complications in the intervention group was 12.20%, which was lower than that in the control group (34.15%) (*p* < 0.05; Table 6).

**Table 6.** Comparison Of incidence of complications between two groups of patients.

	Control group (*n* = 41)	Intervention group (*n* = 41)	χ^2^	*P*
Bleeding	4 (9.76)	2 (4.88)		
Infection	3 (7.32)	1 (2.44)		
Catheter occlusion	2 (4.88)	0 (0.00)		
Hemodialysis-induced hypotension	2 (4.88)	1 (2.44)		
Malnutrition	3 (7.32)	1 (2.44)		
Total incidence rate	14 (34.15)	5 (12.20)	5.549	0.019

## Discussion

Hemodialysis is the most common treatment for ESRF; however, it carries significant risks, including cardiovascular events, hospitalization, and diminished quality of life [19]. This increased demand for hemodialysis not only burdens patients but also places a considerable economic and ecological burden on healthcare systems [20]. Consequently, primary healthcare practitioners must be well-versed in the epidemiology, pathophysiology, and evaluation methods of CKD. In addition, primary healthcare physicians play a crucial role in reducing the risk of CKD and managing the associated complications and comorbidities [21]. Therefore, it is especially important for caregivers to provide professional knowledge support and personalized education to meet patients’ resource and health needs, as well as to promote the proactive use of the various resources available for health promotion. In light of these considerations, our study aimed to observe the impact of the Clark comfortable nursing approach on self-burden and treatment adherence in hemodialysis patients with ESRF.

General self-efficacy is positively associated with quality of life and has the potential to act as a psychological buffer against adverse events and circumstances [22]. In our study, we observed that the intervention group had higher ESCA scores across each dimension as well as the total score compared to the control group. This indicates that providing Clark comfortable nursing interventions can significantly improve self-efficacy levels in patients with ESRF. The primary reason for this is that the nursing model emphasizes self-awareness and self-evaluation, allowing patients to independently choose appropriate problem-solving methods. Throughout this process, patients gain a deeper understanding of themselves, enhance their awareness of the disease, and develop a sense of self-achievement, which ultimately leads to a significant improvement in their self-efficacy levels. However, routine nursing interventions primarily focus on addressing patients’ psychological and emotional states, which can lead to reduced attention to complications or other potential physical issues, resulting in a lack of targeted interventions for root causes.

Psychological disorders and self-perceived burden are common in patients with CKD [23]. Therefore, self-perceived burden should be assessed and managed regularly in the care of patients with ESRF. To this end, the SPBS is commonly used to assess self-perceived burden in patients [24,25]. In this study, after the intervention, both groups showed improvement in various scores and total scores of the SPBS. However, the intervention group had lower scores and total scores compared to the control group, indicating that the Clark comfortable nursing approach can help improve the psychological resilience of long-term hemodialysis patients with ESRF and reduce their self-perceived burden. This improvement could be attributed to the initial implementation of the Clark comfortable nursing approach, where nurses utilized multimedia to educate patients about ESRF, blood dialysis, and the prevention and treatment of complications through pictures, videos, and other formats. This approach facilitates patients’ understanding and retention of relevant knowledge, enhancing their awareness and cooperation.

Adherence to medical advice in hemodialysis patients is crucial for prolonging their life expectancy and improving their quality of life [26]. However, nonadherence to medications is a common and complex problem faced by patients undergoing hemodialysis [27]. Traditionally, routine nursing primarily involves providing health education to patients, explaining the necessity of dialysis treatment and the potential adverse reactions it may cause, and preparing patients psychologically. This is combined with other basic interventions to help patients cooperate with the treatment effectively. However, routine nursing does not sufficiently address the patient’s psychological and physiological needs, resulting in low treatment compliance, which is not conducive to recovery. In contrast, the Clark comfortable nursing approach is based on the core philosophy of fully respecting the patient’s wishes and enhancing their initiative during the nursing period. By strengthening communication with patients, they can become more aware of their own issues and actively seek to address them. This model shifts the focus to supervision and support, which enhances patients’ psychological and physiological well-being.

In this study, the intervention group exhibited higher treatment compliance scores compared to the control group across all measured aspects following the Clark comfortable nursing intervention. Furthermore, both groups demonstrated significant improvements in quality-of-life scores across all dimensions after the intervention. Importantly, the intervention group achieved markedly higher scores in all dimensions after the intervention compared to the control group, demonstrating that the implementation of the Clark comfortable nursing intervention can enhance the quality of life for patients with ESRF. The main reason for this improvement is that the nursing intervention methods encourage patients to actively participate in their care, enabling them to address challenges encountered during the nursing process effectively and autonomously, which enhances their sense of self-responsibility and ultimately promotes better health outcomes.

In summary, the Clark comfortable nursing approach applied to hemodialysis patients with ESRF can improve patients’ self-care abilities, quality of life, and treatment compliance while reducing the incidence of hemodialysis-related complications, making it worthy of clinical promotion. Future research and nursing practice should place greater emphasis on incorporating the concept of comfortable nursing, strengthening nurses’ awareness of self-management education for patients, providing accurate guidance, mobilizing their internal resources, and enabling them to select appropriate management plans based on their individual health conditions. However, this study did not continue to follow up with patients after the nursing intervention ended, nor did we conduct a second psychological state assessment post-dialysis and analyze the results accordingly. Besides, we did not collect blood samples to analyze the effects of blood-related indices in hemodialysis patients, which represents a limitation of our research. In the future, we aim to explore the Clark comfortable nursing approach in greater depth, with the goal of providing stronger data support for the efficacy of treatment in hemodialysis patients with ESRF.
